# Cost Effective Community Based Dementia Screening: A Markov Model Simulation

**DOI:** 10.1155/2014/103138

**Published:** 2014-02-06

**Authors:** Erin Saito, Beau K. Nakamoto, Mario F. Mendez, Bijal Mehta, Aaron McMurtray

**Affiliations:** ^1^Neurology Division, Los Angeles Biomedical Research Institute, 1124 West Carson Street, Torrance, CA 90502, USA; ^2^Neurology Division, Department of Medicine, John A. Burns School of Medicine, University of Hawaii, 1356 Lusitana Street no. 711, Honolulu, HI 96813, USA; ^3^Neurology Department, Straub Clinics and Hospitals, 888 South King Street, Honolulu, HI 96813, USA; ^4^Neurology Department, David Geffen School of Medicine, University of California at Los Angeles, 710 Westwood Plaza, Los Angeles, CA 90095, USA; ^5^Neurology Department, West Los Angeles VA Hospital, 11301 Wilshire Boulevard, Los Angeles, CA 90073, USA; ^6^Neurology Department, Harbor-UCLA Medical Center, Building N-25, 1000 West Carson Street, Torrance, CA 90509, USA

## Abstract

*Background*. Given the dementia epidemic and the increasing cost of healthcare, there is a need to assess the economic benefit of community based dementia screening programs. *Materials and Methods*. Markov model simulations were generated using data obtained from a community based dementia screening program over a one-year period. The models simulated yearly costs of caring for patients based on clinical transitions beginning in pre dementia and extending for 10 years. *Results*. A total of 93 individuals (74 female, 19 male) were screened for dementia and 12 meeting clinical criteria for either mild cognitive impairment *(n* = 7) or dementia *(n* = 5) were identified. Assuming early therapeutic intervention beginning during the year of dementia detection, Markov model simulations demonstrated 9.8% reduction in cost of dementia care over a ten-year simulation period, primarily through increased duration in mild stages and reduced time in more costly moderate and severe stages. *Discussion*. Community based dementia screening can reduce healthcare costs associated with caring for demented individuals through earlier detection and treatment, resulting in proportionately reduced time in more costly advanced stages.

## 1. Introduction

Dementia is a major healthcare problem that causes a significant financial burden to society [1]. Dementia is an age-related progressive decline in cognition that interferes with activities of daily living. This disorder affects about 15% of those over the age of 70 years. If current aging trends continue, the costs of taking care of demented patients may increase by almost 80% by the year 2040 [1]. A recent Rand Corporation study estimates that the total per person cost of dementia care in the US is about $42,000–$69,000/year [1]. Most of this cost is related to caring for patients in the more severe stages of dementia who require institutional and home-based assistance with activities of daily living. Previous reports suggest that early detection leading to early therapy with cholinesterase inhibitors and other treatments can help maintain dementia patients at lower dementia severity levels longer [2, 3]. If so, dementia care costs could be significantly reduced through earlier detection and treatment that maintains patients at less severe stages for a greater portion of the duration of their illness.

Community based dementia screening may be one method that can be used to achieve earlier detection and earlier initiation of therapy, with consequent reduced time spent in more costly higher dementia severity levels. The need for community based dementia screening programs arises from the increasing prevalence of Alzheimer's disease and other progressive dementing disorders in the general population and the consequent rising cost of caring for people with dementia projected to occur in the near future [4]. Challenges to developing effective community based dementia screening programs include factors such as high percentages of inaccurate diagnoses including low sensitivity and low positive predictive values [5], lack of voluntary participation from seniors [6], and the low level of benefit provided by currently available treatment options for Alzheimer's disease which limit the potential benefits from screening programs [7]. Consequently, despite widespread attention given to the rising economic costs of treating and caring for people with Alzheimer's disease and other progressive degenerative dementias [4], a financially cost-effective community based screening program has not yet been demonstrated.

Cadman et al. in 1984 described five essential characteristics needed for an effective community based disease screening program [8]. While these characteristics were originally developed as a guide for infectious disease screening programs, they were worded generally enough to apply equally well to screening programs for other diseases affecting the general population. The five characteristics are paraphrased as follows: (1) to detect a condition with sufficient societal burden, (2) for which treatment options are available, (3) a reliable screening test is available, (4) for which those who could benefit can be reached, and (5) necessary follow-up interventions and monitoring of compliance can be provided [8]. While disease modifying treatment options are still not available for most progressive dementing disorders, we believe that a community based screening program targeting Alzheimer's disease could be developed meeting these criteria and also providing a clear economic benefit to the community.

In this study we report the results of a cost savings analysis using 10-year Markov model simulations for dementia care with and without community based dementia screening. The results of our simulations suggest that implementing an effective community based screening program for dementia could result in up to 9.8% reduction in cost of dementia care over a ten-year period.

## 2. Materials and Methods

### 2.1. Subjects

Participants were adults, over the age of 18 years, who presented sequentially to a community based dementia screening program during a one-year period from January 1st 2011 to January 1st 2012. Deidentified data was obtained and analyzed in a retrospective fashion for all screening program participants. Informed consent was waived since the project consisted of retrospective analysis of already acquired de-identified clinical data.

### 2.2. Dementia Screening Program Description

Screening examinations were conducted by a board certified neurologist in an exam room located within a local community Health Care District office. Community members were made aware of the dementia screening availability via advertisements in the Health Care District monthly newsletter and by word of mouth. Dementia screenings were conducted on one half-day morning per month, with a total of up to 12 fifteen-minute long appointments scheduled per half-day. Each screening evaluation included a standardized workup consisting of a history which included information regarding functional activities and ability to complete activities of daily living independently, physical and neurological examinations, and minimental state examination [9]. Presence of hypertension and diabetes was determined by self-report. Use of tobacco, alcohol, and illicit substances was determined by self-report and categorized “yes” or “no.”

### 2.3. Diagnosis of Cognitive Impairment

Diagnoses of mild cognitive impairment (MCI) and dementia were made by a board certified neurologist according to established clinical criteria [10, 11]. Briefly, participants diagnosed with MCI displayed impairment on cognitive performance as evidenced by MMSE scores below 27 but did not have any impairment on function or activities of daily living noted in their history information, while participants diagnosed with dementia displayed evidence of cognitive impairment with MMSE scores below 25 and also were noted to have impairment in daily activities. Dementia severity was determined by MMSE score, with mild dementia indicated by scores ranging from 21 to 25, moderate dementia by scores ranging from 10 to 20, and severe dementia by scores below 10.

### 2.4. Statistical Analysis

Normally distributed continuous demographic factors and other continuous variables were compared between groups using two-tailed *t*-tests. Nonparametric data was compared between groups using the independent samples Kruskal-Wallis test. Frequency of occurrence of categorical variables was compared between groups using Chi-square analysis or Fisher's exact test as appropriate. All statistical calculations were performed using SPSS version 21 [12].

### 2.5. Markov Model Simulation

Each model simulation included a cohort of 1,000 individuals who were assumed to exist in one of 6 states: nondemented, MCI, mild dementia, moderate dementia, severe dementia, and death. Over a series of ten 1-year cycles patients were allowed to move from one state into a different state based on a predetermined set of transitional probabilities obtained from previously published data as described below [13, 14]. The states used in the model were based on levels of severity of cognitive disability, the model differentiated between detected and undetected individuals with MCI and mild dementia, and an absorbing state of death was included.

At the start of each 1-year cycle, all patients within the model could progress to more severe states of illness, progress to the final state of death, improve to a less severe state, or remain in their current state. Patients with MCI or mild dementia were separated into two categories to differentiate between detected and undetected individuals, each with different transitional probabilities. Patients with moderate-to-severe levels of dementia were assumed to be all detected based on the results of our community based dementia screening program. Transitional probabilities were based on published clinical data from the Consortium to Establish a Registry for Alzheimer's Disease for patients with MCI and with mild, moderate, and severe Alzheimer's dementia [13]. Data from the Alzheimer's Disease Neuroimaging Initiative was used for estimates of progression from predementia to Alzheimer's disease [14].

Each model measured 4 endpoints: total cost per stage and cumulative cost, cost by disease state, time in disease states, and time living. Annual cost estimates were for total cost of care, including both pharmacological and nonpharmacological costs, and were obtained for each disease state using previously published data from the Canadian Study of Health and Aging [15]. Markov model simulations were calculated using TreeAgePro Healthcare version 2013 [16].

## 3. Results

A total of 93 individuals participated in the community based dementia screening program, including 74 females and 19 males (see Table 1). Of the screened individuals, 12 were found to have cognitive impairment (either MCI = 7 or dementia = 5) and 81 were determined to be cognitively normal. Ethnicity distributions matched those reported for the surrounding community, with primarily Caucasian (89.2%) participants, along with some Asian (6.5%) and Hispanic (4.3%) self-reported ethnicities. Compared to cognitively normal participants, cognitively impaired participants displayed greater mean age (mean = 70.32 S.D. = 11.51; mean = 81.83 S.D. = 6.65, resp.; *P* < 0.01) and greater frequency of tobacco use (yes = 9.9% and 25%, resp.; *P* = 0.024). No significant differences were identified for gender or ethnicity distributions, and no significant differences were identified for self-report of alcohol or illicit substance use. A total of 47.5% of the participants reported a family member or friend had been diagnosed with dementia, including 37.6% who reported a family history of a first degree relative with dementia and 12.9% who reported a history of a friend or family member other than a first degree relative who had been diagnosed with dementia. History of family members or friends with dementia did not significantly differ between the groups, although a trend was identified towards a greater frequency of self-report of a friend or family member being diagnosed with dementia among those who were cognitively normal compared to those who were cognitively impaired (yes = 50.6% and 25%, resp.; *P* = 0.0979).

Markov model simulations showed community based dementia screening resulted in a total cost savings benefit over 10 years of $208.54 or 9.83% savings per patient screened compared to no dementia screening (see Table 2). The economic break-even point for the screening program was approximately year 7, with the cost savings occurring in years 8–10 (see Table 2). Community based dementia screening resulted in an increased time spent in MCI and mild dementia states (155% and 247%, resp.) and reduced total percentage time spent in moderate and severe dementia states (32.4% and 35.2% reductions, resp.) (see Table 3). Time spent in the normal cognition and death states were not substantially affected by dementia screening (see Table 3).

**Figure 1:**
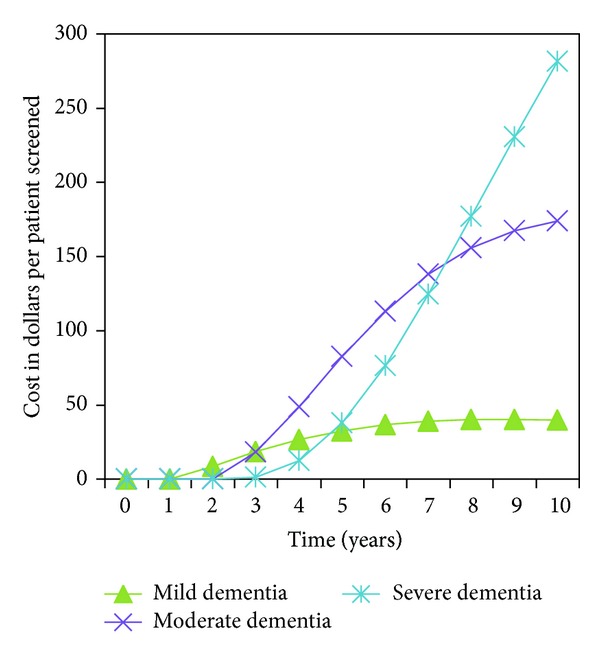
Dementia care costs versus time without dementia screening.

**Figure 2:**
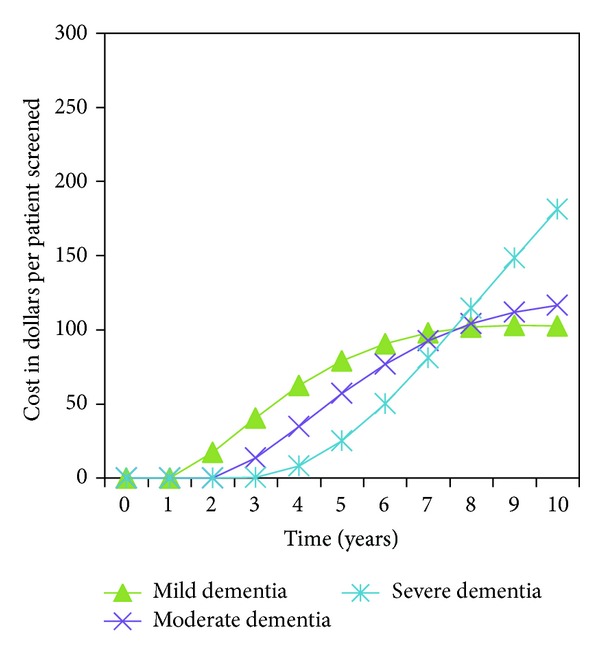
Dementia care cost versus time with dementia screening.

The increased costs associated with dementia screening in years 1 through 6 of the model simulation were attributed to greater rates of detection and treatment of mild dementia during those years compared to care without screening (see Figures 1 and 2). Similarly, the cost savings that occurred during years 8 through 10 were attributed to decreased costs for care of individuals in moderate and severe dementia states due to the decreased time spent in these states with community based dementia screening compared to no screening (See Figures 1 and 2).

## 4. Discussion

Cost of caring for demented individuals places a significant financial burden on both caretakers and society in general. While it is possible that more effective treatments may be developed in the future for progressive dementing disorders, our results suggest that community based dementia screening can achieve a substantial cost savings benefit due to more effective use of currently available treatments through earlier detection and treatment compared to providing care without screening. Overall, our community based dementia screening program met the criteria described by Cadman et al. in 1984 for an effective community disease screening program [8] and demonstrated a cost savings benefit of 9.83% for dementia care related costs over a ten-year period in Markov model simulations compared to standard treatment with no community based screening. We believe the majority of the cost savings likely result from decreased need for caregiver support, achieved by decreasing the average duration spent in more severe disease stages which require more hours of caregiver support compared to less severe disease stages.

The potential cost savings provided by community based dementia screening identified in this study are entirely dependent on the identification of a sufficient number of previously undiagnosed cognitively impaired individuals. In our study we identified new and previously undiagnosed MCI and mild dementia in 7.5% of individuals screened. This rate of detection of previously undiagnosed individuals is in good agreement with previously reported detection rates for community based screening programs which have ranged from 9 to 14% [17, 18]. The ability of community based screening programs to reach previously undiagnosed individuals with dementia has also been demonstrated by Barker et al. in 2005 [19], who showed that subjects with Alzheimer's disease who were referred from a memory screening program presented with higher mean MMSE scores and shorter durations of illness than those referred by physicians or family members.

Others have suggested that it may be possible to increase the rate of detection for previously undiagnosed individuals through enriching the sample by screening individuals deemed to be at high risk for cognitive decline, such as individuals whose functional status has changed; whom friends, family, or caregivers notice a cognitive decline; whom doctors or other health professionals notice signs of cognitive impairment; or even those at advanced ages [3]. If methods such as these are successful in increasing the rate of detection of previously undiagnosed cases through community screening, then this would be expected to result in a directly proportional increase in the cost savings benefit provided by screening programs.

The findings of this study suggest several potential barriers to implementation of community based dementia screening including the use of physician screeners and the lack of voluntary participation of seniors. In our community based dementia screening program seniors utilized 93 of the 144 potentially available screening appointments, resulting in a utilization rate of only 64.6%. Additionally, we experienced particular difficulty engaging male seniors who only comprised 20.4% of the screening participants. Another potential barrier to implementation may result from the greater initial cost to public healthcare systems due to increased cost of treatment resulting from improved identification of MCI and mild dementia cases during the first six years after screening. Particular difficulty may arise due to the long term outlook and planning required for implementing such programs because the benefits of community screening do not become evident until years 8 through 10 after screening takes place.

This study has several limitations. The primary data used in the Markov model simulations was obtained through a cross-sectional study design which limits the value of the information since there is no follow-up data available to determine the reliability or consistency of the diagnoses over time or with further evaluation. Also, the cohort consisted of a convenience sample that was heavily overrepresented by female and Caucasian participants. Consequently the generalizability of the results to males and ethnicities not better represented in the study would require further investigation. Additionally, the diagnosis of dementia was made by a single investigator and consequently the interrater reliability of the diagnosis could not be determined. Another limitation arises from the nature of screening visits which by definition are brief time-limited events and consequently important information such as laboratory test results and brain imaging study results can be lacking.

As rates of dementia rise in the general population, developing cost effective community based dementia screening programs is a topic of increasing importance. The cost savings benefits identified in this study of approximately 10% of dementia care costs over a ten-year period suggest that improved utilization of currently available treatments is sufficient to result in significant savings to local communities. Further study is needed to determine the generalizability of these results to other communities and to improve participation by male seniors. The information obtained in this study may be of use to health care administrators and others interested in developing community based dementia screening programs to reduce costs of providing care to dementia patients.

## Figures and Tables

**Table 1:** Demographic characteristics of memory screening participants.

Characteristic	Cognitively impaired	Cognitively normal	Significance
Mean age in years (standard deviation)	81.83 (6.65)	70.32 (11.51)	<0.01
Gender (female/male)	8/4	66/15	0.257
Ethnicity (Asian/Caucasian/Hispanic)	0/12/0	6/71/4	0.436
Tobacco use (yes/no)	3/9	8/73	0.024
Alcohol use (yes/no)	0/12	11/70	0.604
Illicit substance use (yes/no)	0/12	1/80	1.00
Family history of 1st degree relative with dementia (yes/no)	3/9	32/49	0.235
Family history of dementia other than 1st degree relative (yes/no)	1/11	11/70	0.333
History of friend or non-1st degree family member with dementia (yes/no)	3/9	41/40	0.0979

**Table 2:** Results of Markov model simulation of dementia care costs per participant.

Model stage	No screening	Screening
Year 1	$0	$0
Year 2	$8.676	$17.352
Year 3	$46.650	$72.352
Year 4	$134.583	$177.833
Year 5	$287.622	$339.121
Year 6	$513.904	$556.898
Year 7	$815.167	$828.475
Year 8	$1,188.208	$1,149.078
Year 9	$1,626.475	$1,512.738
Year 10	$2,121.471	$1,912.933

**Table 3:** Cumulative percent time in disease state with and without dementia screening.

Disease state	Cumulative % time with no dementia screening program	Cumulative % time with dementia screening program
Cognitively normal	79.25	79.31
Mild cognitive impairment	2.88	4.46
Mild dementia	0.298	0.737
Moderate dementia	0.349	0.236
Severe dementia	0.256	0.166
Death	20.84	20.82
